# “Non-canonical protein-DNA interactions identified by ChIP are not artifacts”: response

**DOI:** 10.1186/1471-2164-14-638

**Published:** 2013-09-22

**Authors:** Daniel Schindler, Torsten Waldminghaus

**Affiliations:** 1 LOEWE-Center for Synthetic Microbiology (SYNMIKRO), Philipps-Universität Marburg, Hans-Meerwein-Str. 6, D-35043, Marburg, Germany

**Keywords:** ChIP-Chip, ChIP-Seq, False-positive, σ^32^, seqA

## Abstract

**Background:**

Studies of protein association with DNA on a genome wide scale are possible through methods like ChIP-Chip or ChIP-Seq. Massive problems with false positive signals in our own experiments motivated us to revise the standard ChIP-Chip protocol. Analysis of chromosome wide binding of the alternative sigma factor σ^32^ in *Escherichia coli* with this new protocol resulted in detection of only a subset of binding sites found in a previous study by Wade and colleagues. We suggested that the remainder of binding sites detected in the previous study are likely to be false positives. In a recent article the Wade group claimed that our conclusion is wrong and that the disputed sites are genuine σ^32^ binding sites. They further claimed that the non-detection of these sites in our study was due to low data quality.

**Results/discussion:**

We respond to the criticism of Wade and colleagues and discuss some general questions of ChIP-based studies. We outline why the quality of our data is sufficient to derive meaningful results. Specific points are: (i) the modifications we introduced into the standard ChIP-Chip protocol do not necessarily result in a low dynamic range, (ii) correlation between ChIP-Chip replicates should not be calculated based on the whole data set as done in transcript analysis, (iii) control experiments are essential for identifying false positives. Suggestions are made how ChIP-based methods could be further optimized and which alternative approaches can be used to strengthen conclusions.

**Conclusion:**

We appreciate the ongoing discussion about the ChIP-Chip method and hope that it helps other scientist to analyze and interpret their results. The modifications we introduced into the ChIP-Chip protocol are a first step towards reducing false positive signals but there is certainly potential for further optimization. The discussion about the σ^32^ binding sites in question highlights the need for alternative approaches and further investigation of appropriate methods for verification.

## Background

In a recent article in this journal we described o.ur experience with application of the ChIP-Chip method [1]. Our focus was on the replication protein SeqA which had been shown to be specific for hemi-methylated GATC-sequences [2]. To gain a deeper understanding of the DNA-binding of SeqA we applied a widely used standard ChIP-Chip protocol [3]. As a proof that the method works well in our hands we performed ChIP-Chip experiments with RNA-polymerase antibody as published previously [4]. To our great surprise the binding sites we detected for SeqA and RNAP were highly similar. This was absolutely unexpected because many SeqA-bound DNA-regions detected in this experiment did not contain many of the established GATC binding sequences. One possibility is that these non-canonical protein-DNA interactions could be genuine binding sites and therefore an indication that our understanding of DNA-binding proteins is incomplete. We considered the alternative possibility that our surprising results might be artifacts. The key experiment to distinguish between these explanations was a ChIP-Chip using a ΔseqA strain with a SeqA antibody. Also in this experiment we detected binding signals which unambiguously demonstrated unspecifically enriched chromosomal regions via the used standard method. The unspecific signals could be caused by binding of non-target proteins by the antibody. In deed the quality and type of antibody are critical for the quality of ChIP based methods [5,6]. However, the antibody turned out not to be the problem in this case. Evaluation of the ChIP-Chip method led to the identification of four causes for these false signals: i) non-unique sequences, ii) incomplete reversion of crosslinks, iii) inappropriate retention of protein in spin-columns and iv) insufficient RNase treatment [1]. We established a modified ChIP-Chip protocol to minimize the effects of these sources of false positive ChIP peaks and applied it using the SeqA antibody. The SeqA binding pattern detected with this new protocol was radically different from the standard protocol with almost no overlap. This means that specific details of a protocol changed the chromosomal binding pattern completely. The SeqA binding sites we detected with our modified method were exclusively canonical binding sites with binding signals being proportional to the number of GATC sites in the respective regions. Thus, in the case of SeqA the non-canonical protein-DNA interactions identified with the standard ChIP-Chip method are artifacts.

In 2006, Wade and colleagues published a ChIP-Chip study on the alternative sigma factor σ^32^[7]. In addition to 38 known binding sites they surprisingly found 49 new non-canonical binding sites. These non-canonical sites could be either genuine binding sites or artifacts. Wade et al. concluded that these sites are genuine σ^32^ binging sites. Based on our experience with SeqA described above we considered the possibility that these non-canonical sites might instead be false positives. This idea was supported by the lack of a control ChIP-Chip experiment in the Wade et al. study and the fact that they refer to the same protocol that gave the enormous false positive rate in our first SeqA attempt [1]. In our study, the ΔseqA control strain was crucial for identifying false positives. We applied our modified ChIP-Chip protocol to analyze σ^32^ binding on the *E. coli* chromosome. We detected almost all of the canonical σ^32^ binding sites but only very few of the non-canonical sites. Taken together these findings led to the conclusion that the majority of non-canonical σ^32^ sites described by Wade et al. are probably not genuine binding sites but instead false positives [1]. In a recent article in this journal Wade and colleagues published a new study claiming that our conclusion was wrong and that the non-canonical σ^32^ sites are in fact genuine binding sites [8]. They base their view on ChIP coupled with qPCR analyses of 4 out of the 49 “Disputed σ^32^ sites” (DSTs) and the claim that the quality of our ChIP-Chip data is low compared to their study. In addition they find that the specific ChIP enrichment is reduced because of the increased stringency changes we introduced into the protocol. Here, we respond to the new study of the Wade group and use this to discuss some critical questions surrounding ChIP-Chip analysis.

### What is good data quality in ChIP-Chip studies?

As with most methods the quality of ChIP-Chip derived data varies. This might be due to the details of the methodology, the type and quality of the antibody, the biological samples, as well as the performance and experience of the experimenter. Wade and colleagues reanalyzed their own and our data regarding dynamic range and reproducibility and concluded that both aspects were better in their study. We appreciate if other scientists re-analyze our data to come to their own conclusion. This is why we routinely store our ChIP-Chip data in public databases such as the Gene Expression Omnibus (GEO) which is publically accessible. The required detailed description of experimental procedures and data processing together with storage of raw as well as processed data is essential for thorough follow-up analysis. Thus, we recommend the open access storage of genome wide ChIP studies in general. Unfortunately, the debated data of Wade and colleagues are not easily accessible. Wade and colleagues might want to consider storage of their data in a public database to facilitate data comparison and analysis by others. Below, we discuss questions related to the dynamic range and reproducibility of ChIP-Chip derived data.

#### Dynamic range

**Figure 1:**
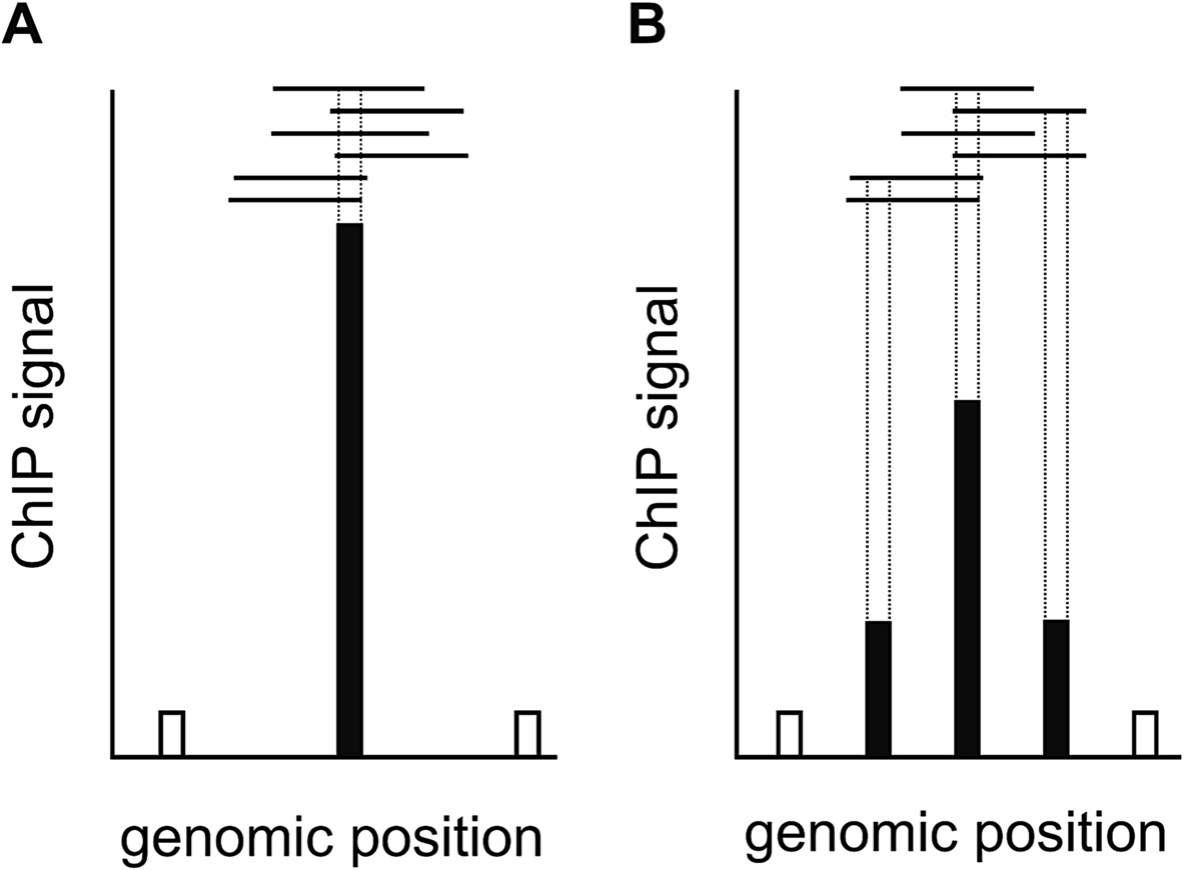
**DNA binding to low and high probe-density microarrays.** On low density microarrays, enriched DNA fragments bind to a single probe **(A)**. This leads to a single strong signal. On high density microarrays the same DNA can bind to more than one specific probe **(B)**. This decreases the signal intensity but increases the number of signals to be used for binding site detection. The dynamic range of raw data will thus be decreased compared to low density microarrays but not the data quality.

We accept the claim by Wade and colleagues that the dynamic range of their study is higher than in our data set. However, the dynamic range is not a suitable quality measurement for inter-platform comparison. The main reason why the dynamic range in our study is lower is because we used an improved microarray with a higher probe number and density. With such a higher probe density the ChIP-DNA is distributed across a greater number of probes (Figure 1). This would certainly decrease the dynamic range but at the same time greatly increase data quality. This is because binding site detection can be assisted by comparisons between multiple neighboring probe signals (Figure 1). Qi and colleagues tested the relationship between probe density and confidence in binding site detection systematically and came to the conclusion that “a single high density microarray (100-bp probe spacing) provides better spatial resolution than three experimental replicates using lower density arrays (300-bp probe spacing)” [9].

If the lower dynamic range of our ChIP-Chip data is the reason why we did not detect the “disputed σ^32^ sites” (DSTs), then this would only apply to the targets with the lowest values. However, the three DSTs with the highest ChIP-score in the Wade et al. paper (*ytfI*, *ygcI* and *yghJ*) were not detected in our study. At the same time we detected known targets that showed a lower score in the Wade et al. study (for example *grpE*, *yccV*, *hepA*).

**Figure 2:**
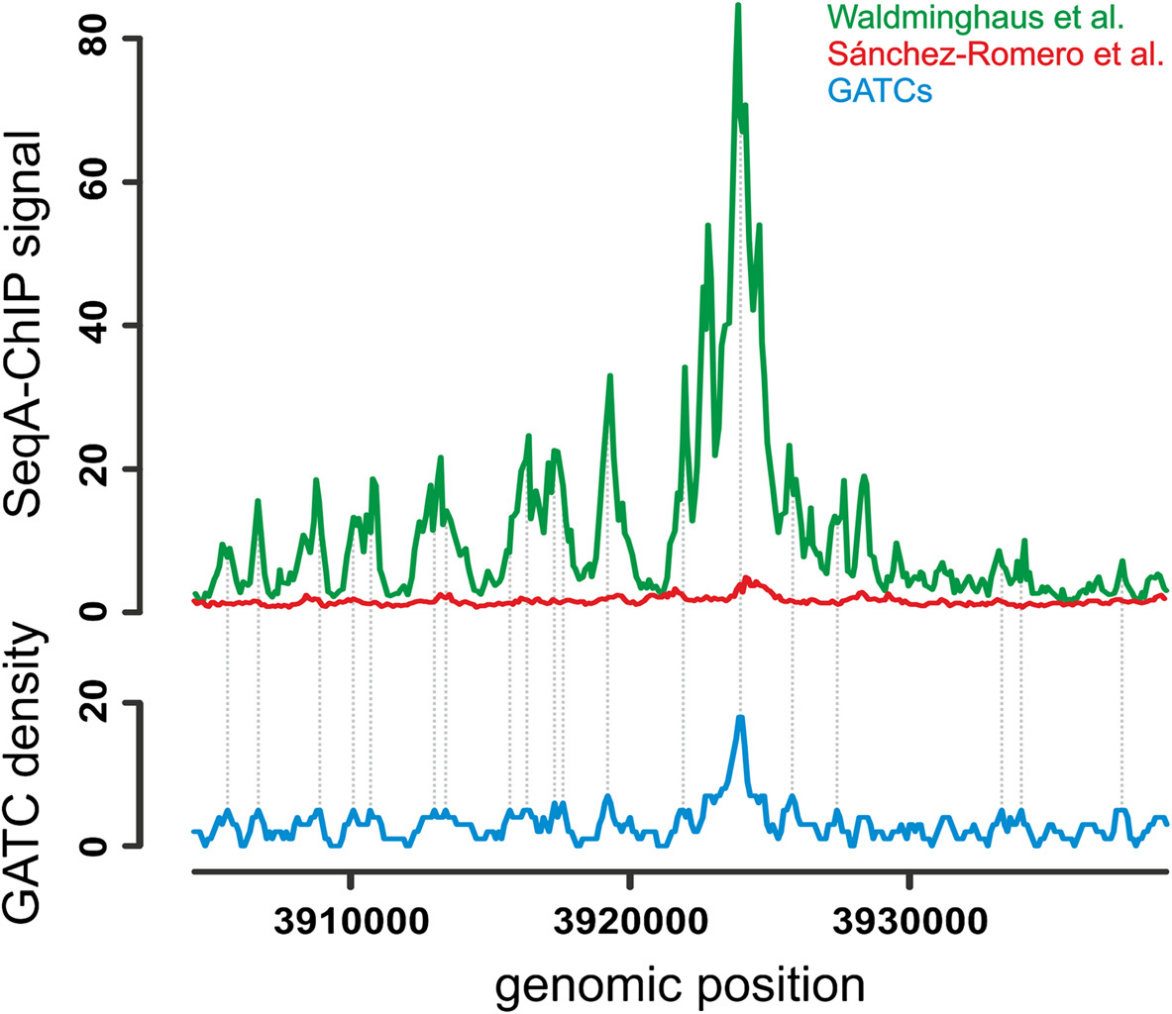
**Comparison of SeqA ChIP-Chip data of Sánchez-Romero et al. **[11]** (red) and Waldminghaus et al. **[10]** (green).** Both studies used *E. coli dnaC2* mutant cells synchronized regarding DNA replication 5 or 6 minutes after initiation and the same antibody for the ChIP reaction. While Sánchez-Romero and colleagues used the original ChIP-Chip protocol, Waldminghaus and colleagues used the modified protocol. While both signal patterns correspond to the GATC density (blue) as expected for SeqA, the dynamic range varies between 5.1 for the Sánchez-Romero et al. experiment and 98.6 for the Waldminghaus et al. experiment. Grey dots show corresponding peaks to a GATC density of ≥ 5 (Moving window of 500 bp; step size 100 bp).

Furthermore, the question remains if our changes to the ChIP-Chip methodology decrease the dynamic range as suggested by Wade and colleagues and whether such a decrease is relevant to this discussion over the identification of false positives. We believe that our modifications of the ChIP-Chip protocol do not prohibit necessary dynamic ranges. Support for this comes from a SeqA ChIP-Chip experiment with synchronized *E. coli* cells [10]. Data was obtained from cells shortly after synchronous initiation of replication (5 or 6 min) using both the standard protocol [11] and our modified version [10]. As Wade and colleagues point out the data from both protocols show similar results (Figure 2). However the dynamic range appears to be higher with our modified protocol (98.6) compared to the study with the standard protocol (5.1; Figure 2). The critical point here is that the same antibody, the same *E. coli* strain and the same microarrays were used for the experiments. Also for genome wide analyses of SeqA binding in unsynchronized *E. coli* cells our changed method gave higher dynamic ranges compared to the standard protocol [1,11].

#### Reproducibility

For reliable data, experiments need to be reproducible and the data from the replicates should be comparable. For ChIP-Chip data, a straight-forward analysis of reproducibility is difficult. This is because most of the data on the microarray can be considered background. Even with a protein of interest binding some hundred times, this will be only a small fraction compared to the whole genome. Subsequently only some probes are expected to give a relevant signal. The remaining probes will detect only background DNA. For calculations of correlation coefficients, as done by Wade and colleagues, this means that one mainly calculates the correlation of the background signal. Thus we consider the information gain of this number limited.

The way we incorporated the reproducibility in our study was to consider only signals as relevant that reached a certain threshold in both replicates, as was the case in the analysis by Wade and colleagues. Since we have in this way detected in our data almost all known and published σ^32^ targets, we consider the reproducibility of our data as solid.

There are other ways to assess the reproducibility of ChIP-Chip and ChIP-Seq data. The critical point is to focus on the target sites. This can be difficult if one lacks an estimate of the expected number of targets. One way to deal with this is a stepwise comparison of ranked target-lists and compute the fraction of overlapping targets in the highest 10, 20, 30, … %. In our previous study we used the highest 1.000 probes (out of 40.000) to plot Venn diagrams for experiment comparison [1]. Such a quantitation of target reproducibility helps the reader in data interpretation and should be provided if possible.

#### Control experiments

**Figure 3:**
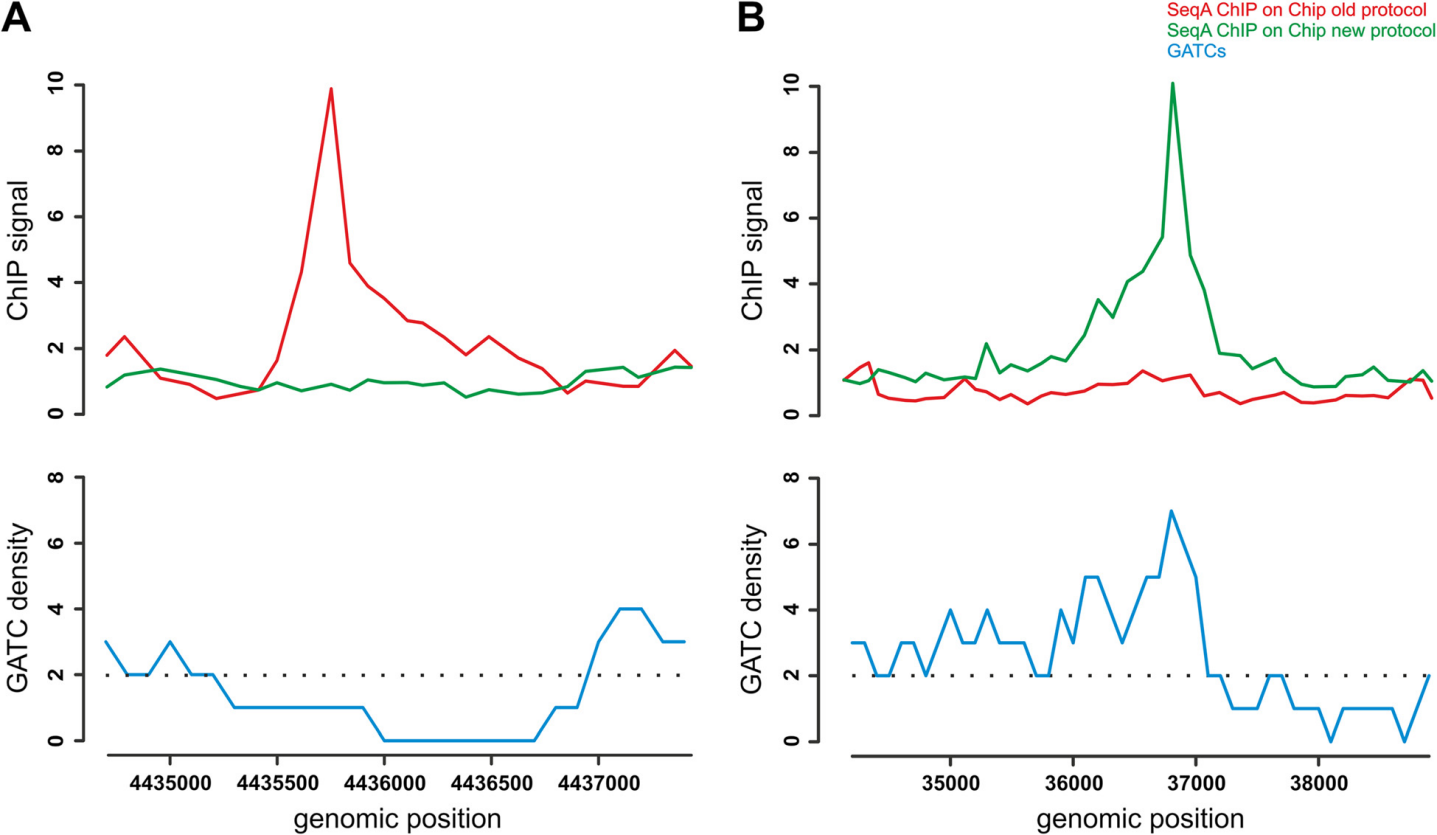
**False positive enrichment peaks resemble true binding sites in ChIP-Chip experiments.** Our modified ChIP-Chip protocol (green) resulted in enrichment data corresponding to the density of GATC binding sites (blue; moving window of 500 bp; step size 100 bp) as expected for SeqA binding with low signals in low GATC regions **(A)** and high signals in GATC dense chromosomal regions **(B)**[1]. The standard ChIP-Chip protocol (red) resulted in erroneous enrichment peaks in regions with low GATC density **(A)** and low signals in GATC rich regions **(B)**. The shape and signal level of the false positive signal example in the *ytfI* gene region **(**red in **A)** and the true positive in the *caiC* gene region **(**green in **B)** are similar.

While discussion about data quality is certainly important, it distracts from the main point of our study. The erroneous data we got for SeqA using the standard protocol had an excellent dynamic range and reproducibility was high. In fact we got the highest dynamic range with our control using a Δ*seqA* strain and the SeqA antibody. However, all of the detected peaks in this experiment must be false. This is actually what we consider the most dangerous fact about the false positive peaks we identified. They appear as wonderful, reproducible hits and not as noise (Figure 3). In our view this is why such false positive enrichments could easily be accepted and published as true binding sites. We have discussed the importance of control experiments as a critical step to identify false positives [1]. Our control experiment for the ChIP-Chip detection of the heat shock sigma factor σ^32^ in heat shocked *E. coli* cells was a similar experiment using non-heat shocked cells. It is remarkable that Wade and colleagues did not include any ChIP-Chip control experiment in their σ^32^ study.

In a recent study, binding of LeuO to the *Salmonella enterica* genome was analyzed by ChIP-Chip [12]. Dillon and colleagues found 261 binding sites using the ChIPOTle peak finding program. However, they were aware of the possibility of false positives in ChIP-Chip data and performed a mock control experiment. In this control 83 peaks were detected overlapping with the 261 potential LeuO peaks. Dillon and colleagues identified them as false-positives and considered only the remaining 178 as likely LeuO binding sites. The approach of Dillon and colleagues supports our argument that, firstly, false positives are a serious problem in ChIP-Chip studies and secondly, control ChIP-Chip experiments can help to detect and reduce false-positives. This is also true for ChIP-Seq where it was shown that peak-scoring algorithms using 2-sample scoring (scoring sample vs. control experiment) perform better than single-sample scoring ones [13].

### Are the new sigma32 targets found by Wade and colleagues real targets or false positives?

Although Wade and colleagues did not include a ChIP-Chip control experiment in their original study, they performed ChIP-qPCR experiments of 3 selected loci out of the 49 “disputed σ^32^ sites” [7]. Notably, here they included a non-heat shock control. In their new study they analyze 3 more loci [8]. The six analyzed loci indeed show temperature dependent association with σ^32^. These results are contradictory to our ChIP-Chip data where no significant temperature dependent association at the respective loci was found. Further experiments might help to resolve this contradiction. It is even more important to analyze the 43 remaining DSTs for which no temperature dependent change in σ^32^ binding has been shown so far. We suggest that alternative methods are needed for verification. Temperature dependent induction of mRNAs at the respective regions could be considered additional evidence but was not found for most “disputed σ^32^ sites” [7,14,15]. Also sequences resembling the well characterized σ^32^ target promoter sequence in the debated regions would promote them as genuine binding sites. However, Wade et al. note that for many of the DSTs no such typical binding sequences could be found [7]. They suggest that at these sites σ^32^ binding is mediated by transcriptional activators that are functional only after heat shock. Identification of these predicted factors would certainly be important for the discussion about DSTs One possibility to find these factors would be mChIP, where proteins co-purified in a ChIP reaction are analyzed [16].

What would be an appropriate alternative method to clarify disputed ChIP sites? For protein interactions, a popular approach is to compliment one pull down experiment with the reverse pull down, meaning both protein partners should be interchangeable as 'bait’ and 'prey’. For ChIP experiments the reverse approach would be to use the DNA as bait to catch the proteins which are proposed to bind this motif. Such methods have actually been developed [17,18].

### Can the ChIP protocol still be improved?

One thing that becomes clear from both our study and that of the current Wade study is that the experimental details can change the output of ChIP-Chip experiments dramatically [1,8]. We did introduce some changes to the method that completely changed the detected SeqA binding pattern towards what we believe to be a more reasonable result. However, we also believe that there is still room for improvements. One main point in consideration is the use of Spin-X columns for washing of the IP bound to Protein A agarose beads. We had found that a problem is the unspecific binding of highly transcribed and consequently highly crosslinked pieces of DNA to the column matrix. We suggest that omission of such columns solves the problem that these unspecific bound fragments are washed off the column in the elution step and appear as peaks on the microarray. Instead of using the columns for collection of the agarose beads we use simple centrifugation and supernatant removal. Wade and colleagues make the point that the columns are necessary to achieve thorough washing. Interestingly they actually omit the columns in the first step of the procedure where the beads are separated from the cell extract [8]. While in the original method description this is done using the Spin-X columns [7], Wade and colleagues collect the beads by centrifugation without columns in this first step just as we suggested to do [8]. This first step is probably the point where most unspecific binding to the column occurs and the omission of columns in this step would be expected to greatly facilitate a reduction in false signals. The following washing steps might be less critical in this respect and the use of Spin-X columns possible or even beneficial. This is certainly a point for further investigations. A related potential improvement is the choice of the actual column to be used. The Spin-X column, for example, is available with various matrix material and pore sizes. We suspect that if unspecific binding to the column is a problem, then this should vary with the pore size and DNA fragment size.

It is noteworthy that other aspects of ChIP-based methods need to be considered beyond the aspects covered by the current discussion. Most prominent is the computational part of the process which provides new challenges with the advent of ChIP-Seq [9,13]. This computational aspect is certainly important for identifying false positives.

## Conclusions

ChIP-Chip or ChIP-Seq are wonderful methods to get insights into protein binding to genomes. We try to promote these methods by optimizing them and alerting other scientists to potential difficulties in data generation and interpretation. We agree with Wade and colleagues that surprising non-canonical protein-DNA interactions can “indicate novel functions for well-studied proteins”. Examples show that non-canonical binding sites can indeed be functional relevant [19,20]. However, we and many others have detected false positives in ChIP-Chip experiments and it is not unlikely that some false positives have not been recognized as such but interpreted and published as real targets. Wade and colleagues write in their conclusion that our view that surprising ChIP-Chip results are often artifacts is a “dogmatic approach” [8]. Our conclusions in that regard were not meant to be taken as dogmatic, but rather a respectful caution against wasteful scientific pursuit that could be based upon erroneous conclusions. The revision of methods and criticism of published results of peers is not always appreciated, and neither is the prospect of having one’s own conclusions questioned. However, it is an essential part of scientific progress. We hope that other scientists examine the results and argumentations published by the Wade group and ourselves and come to their own conclusions. For the future, we are anticipating new results which we hope will help clarify the debated issues surrounding the ChIP-Chip method.

## Abbreviations

DST: Disputed σ^32^ targets; ChIP: Chromatin immunoprecipitation; ChIP-Chip: Chromatin immunoprecipitation with microarray technology; ChIP-Seq: Chromatin immunoprecipitation with next generation sequencing technology; RNAP: RNA polymerase.

## Competing interests

The authors declare that they have no competing interests.

## Authors’ contributions

TW wrote the paper with input from DS. DS prepared the figures with input from TW. Both authors read and approved the final manuscript.

## Response

By Richard P. Bonocora^1^, Devon M. Fitzgerald^2^, Anne M. Stringer^1^ and Joseph T. Wade^1,2†^

^1^Wadsworth Center, New York State Department of Health, Albany, NY 12208, USA

^2^Department of Biomedical Sciences, University at Albany, Albany, NY 12201, USA

^†^corresponding author: jwade@wadsworth.org

Debate and criticism must be welcomed in any scientific endeavour; however, such debate and criticism must also be based on solid experimental evidence. While Schindler and Waldminghaus have responded to our critique [14] of their earlier paper, we note that their response offers no new experimental evidence or data analysis. Hence, our opinion is unchanged: the disputed sites of σ^32^ binding (DSTs) are genuine, and non-canonical sites identified by ChIP-chip or ChIP-seq are not artifacts to be disregarded. We feel that three points require specific attention:

**1. The modifications to the ChIP method proposed by Waldminghaus and colleagues do not improve data quality.** By directly comparing the two methods in targeted, controlled ChIP assays, we have clearly demonstrated that the standard ChIP method is more effective than the modified method at detecting association of σ^32^ with both well-established targets and DSTs. Additional experiments with the transcription factor AraC confirm that the general outcome of ChIP experiments is unchanged by the use of Spin-X columns during the wash steps; if anything, use of Spin-X columns increases signal. We note that most ChIP studies do not use Spin-X columns until the wash steps, and this modification to the method was applied before the study of Waldminghaus and Skarstad [1], e.g. [21]. Instead of representing a methodological improvement, we propose that Waldminghaus and Skarstad’s σ^32^ ChIP-chip experiments simply had reduced sensitivity due to a combination of the ChIP protocol, antibody, and/or growth conditions, all of which differed from our own.

Although we have focused on σ^32^, an independent study of SeqA, using the standard ChIP-chip method, yielded almost identical data to those generated by Waldminghaus and colleagues. This similarity was recently noted by a group who successfully used the standard ChIP method to measure DNA binding by SeqA [22]. The data presented by Schindler and Waldminghaus in Figure 2A is misleading because the scales are not comparable for the two datasets, and the time-points after replication initiation are different (signal at the replication origin is expected to drop rapidly following replication initiation).

**2. Most, if not all DSTs are genuine sites of σ**^**32 **^**association.** Many lines of evidence support this. First, we tested 6 DSTs using ChIP/qPCR, with an appropriate control; all 6 targets were confirmed. Whilst the remaining 43 DSTs were not tested, our data strongly suggest that most are genuine sites of σ^32^ association. We find it unrealistic to suggest that every single target identified by a ChIP-chip or ChIP-seq experiment should be validated individually. Second, while the standard ChIP protocol reliably yields higher enrichment values at DSTs in targeted experiments, the modified method also detects σ^32^ binding at 2 out of 4 DSTs tested. Moreover, the ranking of DSTs as a group increases significantly among all genomic regions following heat shock in Waldminghaus and colleagues’ ChIP-chip data (Mann-Whitney U Test, *p* = 1e^-6^), as would be expected for genuine sites of σ^32^ association. Both of these findings support the idea that discrepancies between the datasets are related to differences in assay sensitivity, not the validity of targets. Third, nine of the DSTs were identified in independent transcriptomic studies [14,15] and we have shown that RNA polymerase levels increase at two of four DSTs tested following overexpression of σ^32^. These two DSTs were not identified by either transcriptomic study, indicating that such experiments are not necessarily sensitive enough to detect all regulated RNAs. Fourth, DSTs are more likely to be located in intergenic regions than expected by chance (Binomial Test, *p* = 0.00033).

Schindler and Waldminghaus suggest that DSTs are not real σ^32^ binding sites because many of them lack detectable motifs and/or were not detected in transcriptomic studies. However, since DSTs are generally weakly bound, they would be expected to bind more degenerate motifs and be associated with more subtle changes in transcription.

**3. Non-canonical sites for DNA-binding proteins are often real, and studying them is not a wasteful scientific pursuit.** We reassert that the headline conclusion of Waldminghuas and Skarstad’s paper “ChIP on Chip: surprising results are often artifacts”, and their subsequent criticism of work from multiple laboratories, is highly misleading. We and others have identified many non-canonical targets for bacterial DNA-binding proteins that have been validated in controlled experiments. In fact, 15 of the 47 σ^32^ targets identified by Waldminghaus and Skarstad are non-canonical (located inside genes or not associated with detectable regulation in transcriptomic studies) and, by their own logic, should be mistrusted. Although ChIP-chip and ChIP-seq studies in bacteria have been limited in number, a recent large-scale ChIP-seq study in *Mycobacterium tuberculosis* identified hundreds of non-canonical transcription factor binding sites [23]. We conclude that non-canonical binding sites for bacterial DNA-binding proteins occur often and should be the subject of further study precisely because they do not conform to the text-book model of transcription regulation.

ChIP-chip data from our original study [7] are now publicly available from the EMBL-EBI ArrayExpress database (E-MTAB-1849).
